# lncRNA KRAL reverses 5-fluorouracil resistance in hepatocellular carcinoma cells by acting as a ceRNA against miR-141

**DOI:** 10.1186/s12964-018-0260-z

**Published:** 2018-08-17

**Authors:** Lili Wu, Chenwei Pan, Xin Wei, Yifen Shi, Jianjian Zheng, Xiangyang Lin, Liang Shi

**Affiliations:** 1 Department of Clinical Laboratory, The central hospital of Wenzhou, The Dingli Clinical College of Wenzhou Medical University, Wenzhou, China; 2 Department of Infectious Disease, The Second Affiliated Hospital and Yuying Childrens Hospital of Wenzhou Medical University, Wenzhou, China; 3 The First Clinical College and The First Affiliated Hospital of Wenzhou Medical University, Wenzhou, China; 4 Department of Hematology, The First Affiliated Hospital of Wenzhou Medical University, Wenzhou, China; 5 Key Laboratory of Diagnosis and Treatment of Severe Hepato-Pancreatic Diseases of Zhejiang Province, The First Affiliated Hospital of Wenzhou Medical Uinversity, Wenzhou, China; 6 Department of Laboratory Medicine, The First Affiliated Hospital of Wenzhou Medical Uinversity, Wenzhou, China

**Keywords:** Long non-coding RNA (lncRNA), miR-141, Keap1, 5-fluorouracil, Chemoresistance, Hepatocellular carcinoma

## Abstract

**Background:**

5-Fluorouracil (5-FU) has been widely applied to treat various types of cancers, including hepatocellular carcinoma (HCC). However, primary or acquired 5-FU resistance prevents the clinical application of this drug in cancer therapy. Herein, our study is the first to demonstrate that lower expression of KRAL, a long non-coding RNA (lncRNA), mediates 5-FU resistance in HCC via the miR-141/Keap1 axis.

**Methods:**

Cell proliferation assays, western blot analysis, qRT-PCR, the dual-luciferase reporter assay and RNA immunoprecipitation were performed to investigate the mechanisms by which KRAL mediates 5-fluorouracil resistance in HCC cell lines.

**Results:**

The quantitative analysis indicated that KRAL and Keap1 were significantly decreased and that Nrf2 was increased in HepG2/5-FU and SMMC-7721/5-FU cells compared with the corresponding expression levels in the respective parental cells. Overexpression of KRAL increased Keap1 expression, and inactivating the Nrf2-dependent antioxidant pathway could reverse the resistance of HepG2/5-FU and SMMC-7721/5-FU cells to 5-FU. Moreover, KRAL functioned as a competitive endogenous RNA (ceRNA) by effectively binding to the common miR-141 and then restoring Keap1 expression. These findings demonstrated that KRAL is an important regulator of Keap1; furthermore, the ceRNA network involving KRAL may serve as a treatment strategy against 5-FU resistance in hepatocellular carcinoma cells.

**Conclusions:**

KRAL/miR-141/Keap1 axis mediates 5-fluorouracil resistance in HCC cell lines.

## Background

Hepatocellular carcinoma (HCC) is the fifth most common malignancy, causing over 600,000 deaths annually worldwide. Despite the fact that chemotherapy and aggressive treatments have been developed, the 5-year survival rate for HCC is still low because of late diagnosis, tumour relapse, and drug resistance. Chemoresistance is the major cause for the failure of cancer therapy and is still a profound challenge for clinical treatment [1]. Therefore, deciphering the molecular mechanisms underlying chemoresistance in HCC, especially the genetic and epigenetic alterations, is an urgent focus for HCC chemotherapies.

The Kelch-like ECH-associated protein 1 (Keap1)-nuclear factor erythroid 2-related factor 2 (Nrf2) signalling axis [2] acts as “cellular defensive machinery” in response to oxidative/electrophilic stimuli and chemical insults. Keap1 serves as a substrate adaptor protein between Nrf2 and the ubiquitin ligase Cullin-3 (Cul3) and accelerates proteasomal Nrf2 degradation. However, the modification of specific thiols hampers Keap1-mediated proteasomal degradation; therefore, Nrf2 is released from Keap1 and translocates into the nucleus, resulting in subsequent transactivation of a wide array of downstream genes involved in the metabolism and detoxification of free radicals. Several groups demonstrated that constitutive activation of Nrf2 promotes tumour cell growth and survival, and this “dark” side of Nrf2 conferred chemo- and/or radio-resistance during anti-cancer therapies [3].

Long non-coding RNAs (lncRNAs), which are defined as non-coding RNAs longer than 200 nucleotides, participate in diverse cellular processes, including cellular proliferation, differentiation, migration, invasion, apoptosis, alternative splicing and miRNA sponging. Recent studies have suggested that several aberrantly expressed lncRNAs mediate drug resistance [4]. TUG1, for example, has been reported to mediate MTX resistance in CRC cells via the miR-186/CPEB2 axis [5]. MDR1 expression could be enhanced by the lncRNA H19, thus promoting doxorubicin accumulation and increasing acceptable toxicity levels in HCC cells [5, 6]. The overexpression of the lncRNA PVT1 could enhance MDR1 expression and resistance to the pro-apoptotic activity of cisplatin in gastric cancer cells [7]; however, the role of lncRNA in Keap1 regulation and 5-fluorouracil (5-FU) resistance of HCC remains unclear. In the present study, we analysed the expression profile of lncRNAs in 5-FU resistant HCC cells and their counterpart cells, Among the differentially expressed lncRNAs, ENST000004977918 was observed to be located 412 kb from the Keap1 gene on chromosome 19q10.14. Because this lncRNA may regulate Keap1 gene expression, it was named Keap1 regulation-associated lncRNA (KRAL). After its initial discovery as an important modulator of Keap1 expression, further investigation demonstrated that ectopic expression of KRAL could sequester miR-141 to upregulate Keap1 expression, repressing the Nrf2-dependent antioxidant pathway and thus reversing the resistance of HCC cells to 5-FU. Taken together, these results demonstrated that KRAL reverses 5-FU resistance by acting as a ceRNA against miR-141 in HCC cells.

## Methods

### Cell culture

The HCC cell line HepG2 was obtained from the Academy of Military Medical Science of the PLA (Beijing, China) and cultured in Dulbecco’s Modified Eagle’s Medium (SH30249.01, HyClone, USA). SMMC-7721 cells were maintained in RPMI 1640 (21,875,034, Thermo Fisher Scientific). All culture media were supplemented 10% foetal bovine serum (FBS, Gibco BRL, Grand Island, NY), and cells were incubated at 37 °C in a humidified atmosphere containing 5% CO_2_. 5-FU-resistant HepG2 and SMMC-7721 cells were developed as previously described [8]. The drug-resistant phenotypes were maintained in drug-free medium for 2 weeks prior to experimentation. Cells in the logarithmic growth phase were used in all the experiments.

### Cell viability

Cells were seeded into 96-well plates at an initial density of 4 × 10^3^ cells per well. After 12 h of incubation, cells were incubated in fresh culture medium containing different concentrations of 5-FU for 48 h. The cell counting kit 8 (CCK-8; CK04–100,Dojindo, Kumamoto Prefecture, Kyushu, Japan) assay was performed to analyse cell viability. The absorbance of the resulting coloured solution at 450 nm was measured using a spectrophotometer. All experiments were performed in triplicate.

### Microarray analysis

An array analysis platform (Agilent Technologies, Santa Clara, USA) was used for microarray analysis. Briefly, purified mRNAs were amplified and transcribed into double-stranded complementary DNA (cDNA), which was labelled and hybridized onto a Human LncRNA Array v3.0 according to the manufacturer’s instructions as described previously [8], Raw data was normalized and adjusted using the GenePix Pro 4.0 software. The student’s *t*-test analyses were carried out between HepG2 and HepG2/5-FU samples, and lncRNAs with *p* values of < 0.05 were chosen for cluster analysis using a hierarchical method and average linkage and Euclidean distance metric.

### Human specimens

In all, 30 HCC tissues were obtained from the First Affiliated Hospital of Wenzhou Medical University between 2016 and 2017. No patients received preoperative radiotherapy or chemotherapy prior to tissue resection. HCC was diagnosed according to the WHO classification system by three pathologists. Tumour specimens were snap-frozen in liquid nitrogen and stored at − 80 °C immediately after resection. This study was approved by the Ethics Committee of the First Affiliated Hospital of Wenzhou Medical University, and written informed consent was received from all patients prior to tissue resection.

### Western blotting

Whole cell and nuclear lysates were prepared as described previously [9]. The Bradford method (Thermo) were performed to detect the protein concentrations, Approximately 30 μg of protein was loaded onto gels for sodium dodecyl sulphate-polyacrylamide gel electrophoresis (SDS-PAGE) and then transferred onto a nitrocellulose membrane (Bio-Rad), which were incubated with primary antibodies (Keap1, 1:2000, ab139729, abcam, UK; Nrf2, 1:3000, sc-365,949, Santa Cruz, CA; HO-1, 1:1000, sc-103,492, Santa Cruz, CA; GAPDH, 1:6000, sc-20,358, Santa Cruz, CA) and visualized by an enhanced chemiluminescence kit (Roche).

### RNA isolation and real-time PCR

Total RNA was extracted from cancer cells with TRIzol reagent according to the manufacturer’s instructions. First-strand cDNA synthesis was performed by using a PrimeScript 1st Strand cDNA Synthesis Kit (RR014A, Takara). The synthesized cDNA template was added to SYBR Green Mix (04913850001, Roche) for real-time PCR (RT-PCR) with a 7500 Real-Time PCR System (Applied Biosystems, USA). To detect miR-141 expression, the amount of U6 mRNA was used to normalize transcriptional quantification, which was performed with the 2^-ΔΔCt^ method. For KRAL and Keap1 mRNA expression analysis, GAPDH served as an internal control.

### Plasmid construction

Overexpression or knockdown of KRAL was performed with a lentiviral system. For knockdown plasmids, the shRNA sequences targeting KRAL or scramble shRNA was annealed and cloned into the pLKO.1 vector. The target sequences of KRAL were as follows: sh1:5’CCAGGAAGTCCCACATATA3’, and sh2: 5’AACTCATGCCACCTCATCA3’. pLV vectors were ligated with KRAL containing target sequences for hsa-miR-141. Lentiviral particles expressing the above shRNAs or KRAL were produced in HEK293T cells, transfected into cells for 48 h and then selected with 1 mg/mL puromycin for 4 days. To construct luciferase reporter plasmids, Keap1–3’-UTR, Keap1–3’-UTR-mut (mutations in the miR-141 binding sites), wild-type KRAL cDNA or KRAL cDNA containing mutations at the miR-141 binding sites were amplified and subcloned downstream of the luciferase gene in the pmirGLO reporter vector. These plasmids were named as pmirGLO-Keap1 (or mut) and pmirGLO-KRAL (or mut), respectively.

### Dual-luciferase reporter assay

For the luciferase assay, cells (1.5 × 10^5^) were grown in a 24-well plate and were co-transfected with 100 ng of either miR-141 mimics or negative control, 30 ng of firefly luciferase plasmids containing either the wild-type or mutant KRAL fragment, Keap1, and 2 ng of pRL-TK (Promega, Madison, WI, USA) using Lipofectamine 3000 (Invitrogen) according to the manufacturer’s protocol. At 48 h after transfection, the luciferase activity in the cells was measured using a luciferase assay kit (Promega) and normalized to the Renilla luciferase activity for each transfected well. Independent experiments were performed in triplicate.

### siRNA transfection

Chemically synthesized Keap1-specific siRNA (5’GAATGATCACAGCAATGAA3’) was obtained from Shanghai GenePharma Co., Ltd. Cells in the logarithmic growth phase were transfected with Keap1 siRNA or scrambled siRNA using Lipofectamine 3000 and HiPerFect (Invitrogen) Transfection reagent according to the manufacturer’s instructions.

### RNA immunoprecipitation

The RNA immunoprecipitation (RIP) assay was performed using an EZMagna RIP kit (Millipore, Billerica, MA, USA) according to the manufacturer’s protocol. In brief, cells were co-transfected with pMS2-GFP (27,121, Addgene) and pLV-MS2, pLV-KRAL-MS2, or pLV-KRAL-mut-MS2 (mutations in the miR-141 binding sites). After 48 h, cells were harvested for the RIP assays by using a Magna RIP™ Kit (Millipore) according to the manufacturer’s protocol. Cells were lysed in complete RIP lysis buffer containing protease and RNase inhibitors. Magnetic beads were incubated with an anti-GFP (ab13970, abcam) or anti-rabbit IgG (AB5711, Millipore) antibody at 25 °C for 2 h, and whole-cell extracts were immunoprecipitated with the antibody-treated beads at 4 °C overnight. RNA bound to protein was isolated and detected by qRT-PCR using respective primers to quantify the presence of the binding targets.

### Statistical analyses

The data in triplicate are expressed as the mean ± standard deviation (SD). Comparisons between two groups were evaluated using Student’s t-test, and comparisons between multiple groups were evaluated using one-way ANOVA. Correlation between miR-141 expression and KRAL level in 30 HCC tissue samples were evaluated by Pearson’s correlation coefficient. All statistical analyses were performed with GraphPad Prism 7.0 software (GraphPad Software Inc., USA). A *p* value less than 0.05 was considered statistically significant.

## Results

### KRAL expression is downregulated in 5-fluorouracil-resistant HCC cells

**Fig. 1:**
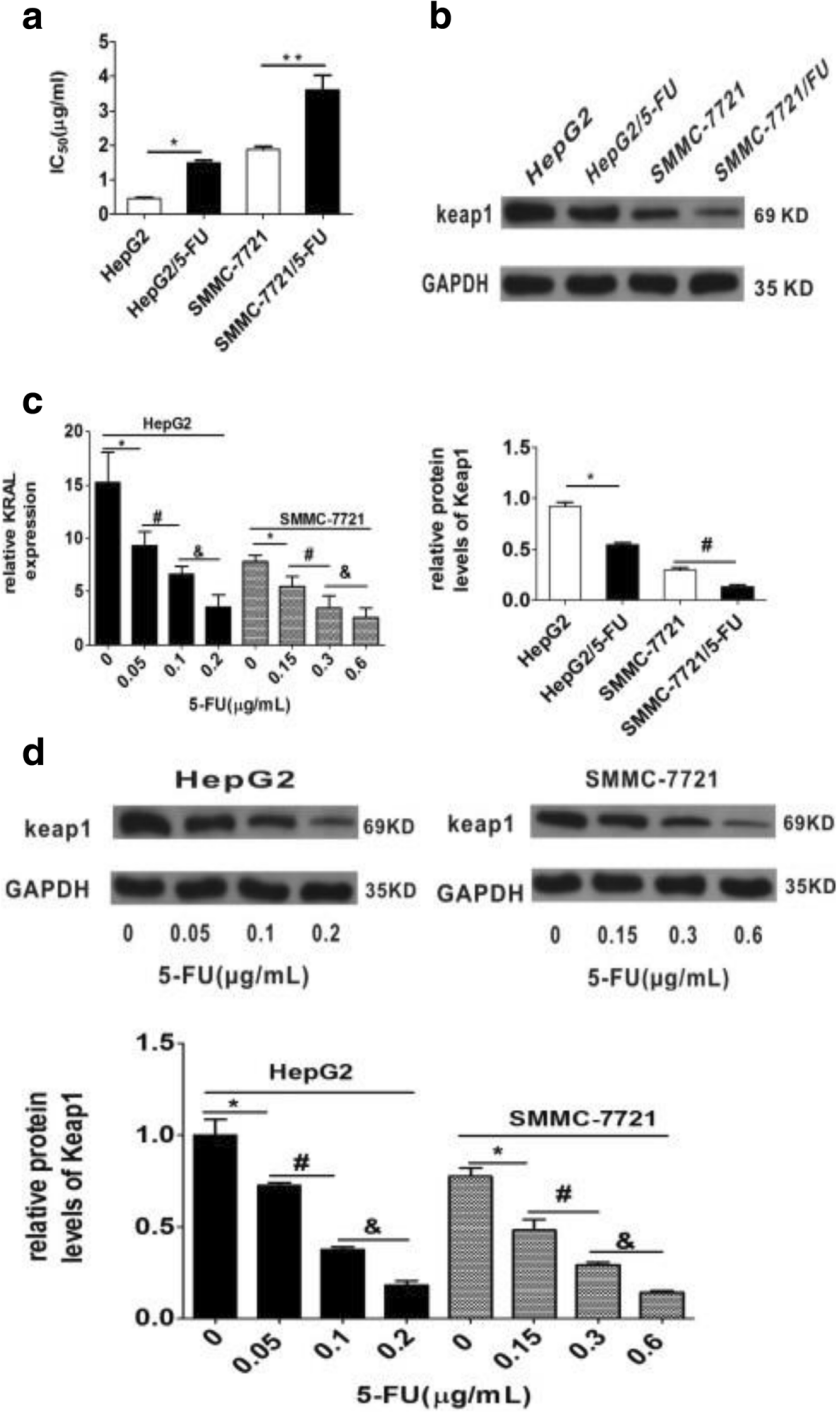
Downregulation of KRAL is associated with 5-fluorouracil resistance in HCC cells. **a** The IC_50_ values of 5-FU in HepG2, SMMC-7721, HepG2/5-FU, and SMMC-7721/5-FU cells. Data are expressed as the mean ± SD; columns: mean of three independent experiments; *p < 0.05, **p < 0.01. **b** The protein levels of Keap1 in HepG2, SMMC-7721, HepG2/5-FU, and SMMC-7721/5-FU cells were detected by western blot. GAPDH was used as a reference, data are expressed as the mean ± SD; the bar graph indicates the normalized values from at least 3 separate experiments; *p < 0.05, ^*#*^*p* < 0.05 vs the respective parent cells. **c** qRT-PCR was performed to detect the relative expression of KRAL in HepG2 and SMMC-7721 cells treated with different non-cytotoxic doses of 5-FU, GAPDH was used as a reference, Data are expressed as the mean ± SD; columns: normalized mean values of three independent experiments; *p < 0.05, ^*#*^*p* < 0.05, ^&^*p* < 0.05. **d** The protein level of Keap1 in HepG2 and SMMC-7721 cells treated with different non-cytotoxic concentrations of 5-FU was analysed by western blot. GAPDH was used as a reference; data are expressed as the mean ± SD; the bar graph indicates the normalized values from at least 3 separate experiments; *p < 0.05 vs the 0 μg/mL group, ^#^*p* < 0.05 vs the 0.05 μg/mL or 0.15 μg/mL group, ^&^*p* < 0.05 vs the 0.1 μg/mL or 0.3 μg/mL group

**Fig. 2:**
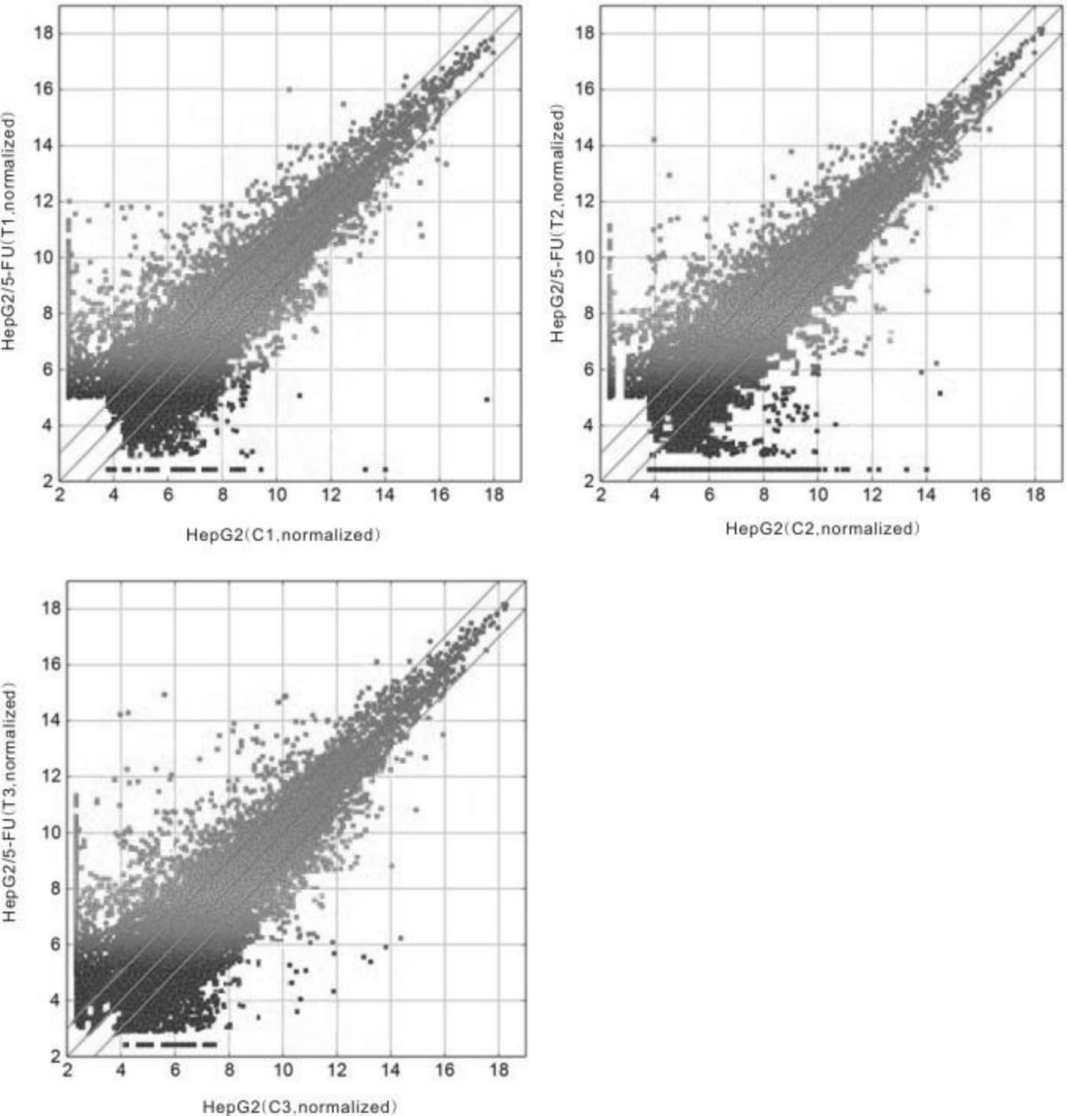
scatter plots were used for quality assessment of differentially expressed lncRNAs between HepG2/5-FU and HepG2 arrays. The averaged normalized values were shown in each group (log 2-scaled). The lncRNAs above the top line and below the bottom line are those with a > 2.0-fold change in expression between HepG2/5-FU and HepG2 arrays. The experiment was repeated three times

**Fig. 3:**
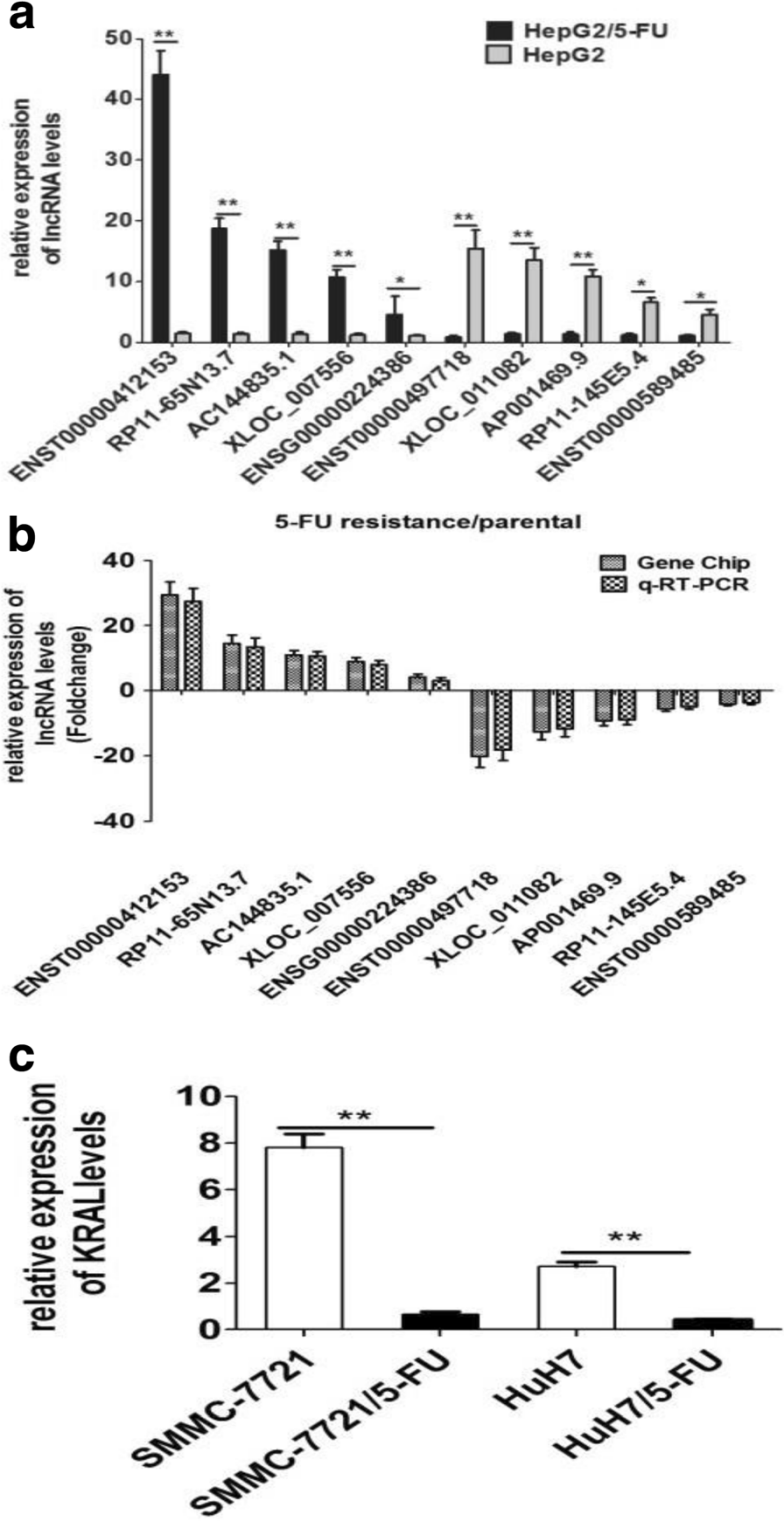
KRAL expression is downregulated in 5-fluorouracil-resistant HCC cells. **a** Comprehensive panel of ten differentially expressed lncRNAs in HepG2/5-FU and HepG2 cells were validated by qRT-PCR. GAPDH was used as a reference, Data are expressed as the mean ± SD; columns: normalized mean of three independent experiments; *p < 0.05, **p < 0.01. **b** A comparison between the microarray Gene Chip and RT-PCR data in the expression (fold change, 5-FU resistance/parental) for differentially expressed lncRNAs. Columns: fold change of three independent experiments. **c** qRT-PCR was performed to detect the relative expression of KRAL in SMMC-7721, SMMC-7721/5-FU, HuH7, and HuH7/5-FU cells. GAPDH was used as a reference, Columns: normalized mean of three independent experiments; **p < 0.01

To identify lncRNAs associated with 5-FU resistance in HCC cells, the 5-FU resistant cell line HepG2/5-FU and SMMC-7721/5-FU were constructed based on established protocols. The IC_50_ values of 5-FU in HepG2/5-FU and SMMC-7721/5-FU cells was much higher than those in their respective parent cells as previously described (Fig. 1a). An lncRNA microarray analysis was performed between HepG2/5-FU and their parental HepG2 cells. In total, 3086 lncRNAs were differentially expressed in HepG2/5-FU cells, including 1762 upregulated and 1324 downregulated lncRNAs (fold change ≥2.0, *p* < 0.05, Fig. 2), compared with those in HepG2 cells. Among these differentially expressed lncRNAs, lncRNA KRAL was downregulated more than twenty-fold in HepG2/5-FU cells compared with the levels in parental HepG2 cells (Table 1). Next, 5 significantly up- and 5 downregulated lncRNAs (Tables 1 and 2) were chosen and verified by qRT-PCR in HepG2 and HepG2/5-FU cells to confirm the results of the microarray data (Fig. 3a-b). We also observed a − 18.5-fold and − 6.3-fold change in the KRAL mRNA expression levels in SMMC-7721/5-FU and HuH7/5-FU cells, respectively, compared with the levels in their respective parental cells (Fig. 3c). This result suggests that KRAL downregulation may play a key role in the mechanism of 5-FU.

**Table 1:** Comparison of ten differentially expressed lncRNAs in 5-FU-resistant HepG2 cell lines and their parental counterparts

Name	Chromosome	Regulation	Fold change	*P* value
ENST00000412153	2	Up	29.38	0.002
RP11-65 N13.7	9	Up	14.37	0.005
AC144835.1	15	Up	10.87	0.007
XLOC_007556	9	Up	8.91	0.003
ENSG00000224386	3	Up	4.11	0.020
ENST00000497718	19	Down	20.32	0.004
XLOC_011082	14	Down	12.67	0.006
AP001469.9	21	Down	9.17	0.009
RP11-145E5.4	9	Down	5.51	0.037
ENST00000589485	11	Down	4.11	0.042

**Table 2:** Primer sequences for real-time PCR

LncRNA	Forward and Reverse Primer	Product length
ENST00000412153	CTGCAGACTTGCTCTTTGTACC	380
	ATGCTCCCATACTCCACTCC	
RP11-65 N13.7	CCTGGGCTCAATCAATCCTT	213
	ATTCCAGCACTTTGGGAAGC	
AC144835.1	TGGCTTAGTTAGACCAACCG	177
	CCAGCTTTCCCTCCAATCAC	
XLOC_007556	AGGAGGGTAAGGCAGGAGAAT	104
	TAAGAGTCTCGCTCTGTCACCC	
ENSG00000224386	ATCCCTGGAATGAGGCACC	121
	TTCCAGGCTCTGAGGCAACT	
ENST00000497718	CCAGTGGACGGACATGCTTT	295
	CACAGAGTTTGTGAGGGAGT	
XLOC_011082	AGGACAGGACCACTGATAAGCC	229
	CTCAAAGTGCTGGGATTACAGG	
RP11-145E5.4	AGAAAGGAAAGCGAGGTCAT	233
	CCTTTGAAATGTCGTGGC	
AP001469.9	ATACAATCACTTCCCACCAG	376
	TGACAACAAAGCAAGACCCT	
ENST00000589485	TGGCTTAGTTAGACCAACCG	177
	CCAGCTTTCCCTCCAATCAC

### Downregulation of KRAL is associated with 5-fluorouracil resistance in HCC cells

Keap1 expression was detected by western blot in HepG2, SMMC-7721, HepG2/5-FU and SMMC-7721/5-FU cells. It was discovered that Keap1 expression in HepG2/5-FU and SMMC-7721/5-FU cells is lower than that in their respective parent cells (Fig. 1b). As decreased KRAL expression is negatively associated with augmented 5-FU resistance, it has been hypothesized that 5-FU may lead to the downregulation of KRAL. HepG2 and SMMC-7721 cells were treated with different non-cytotoxic doses of 5-FU, and we found that 5-FU decreased KRAL expression in a dose-dependent manner (Fig. 1c). Keap1 expression also showed a similar trend (Fig. 1d).

### Ectopic expression and knockdown of KRAL

**Fig. 4:**
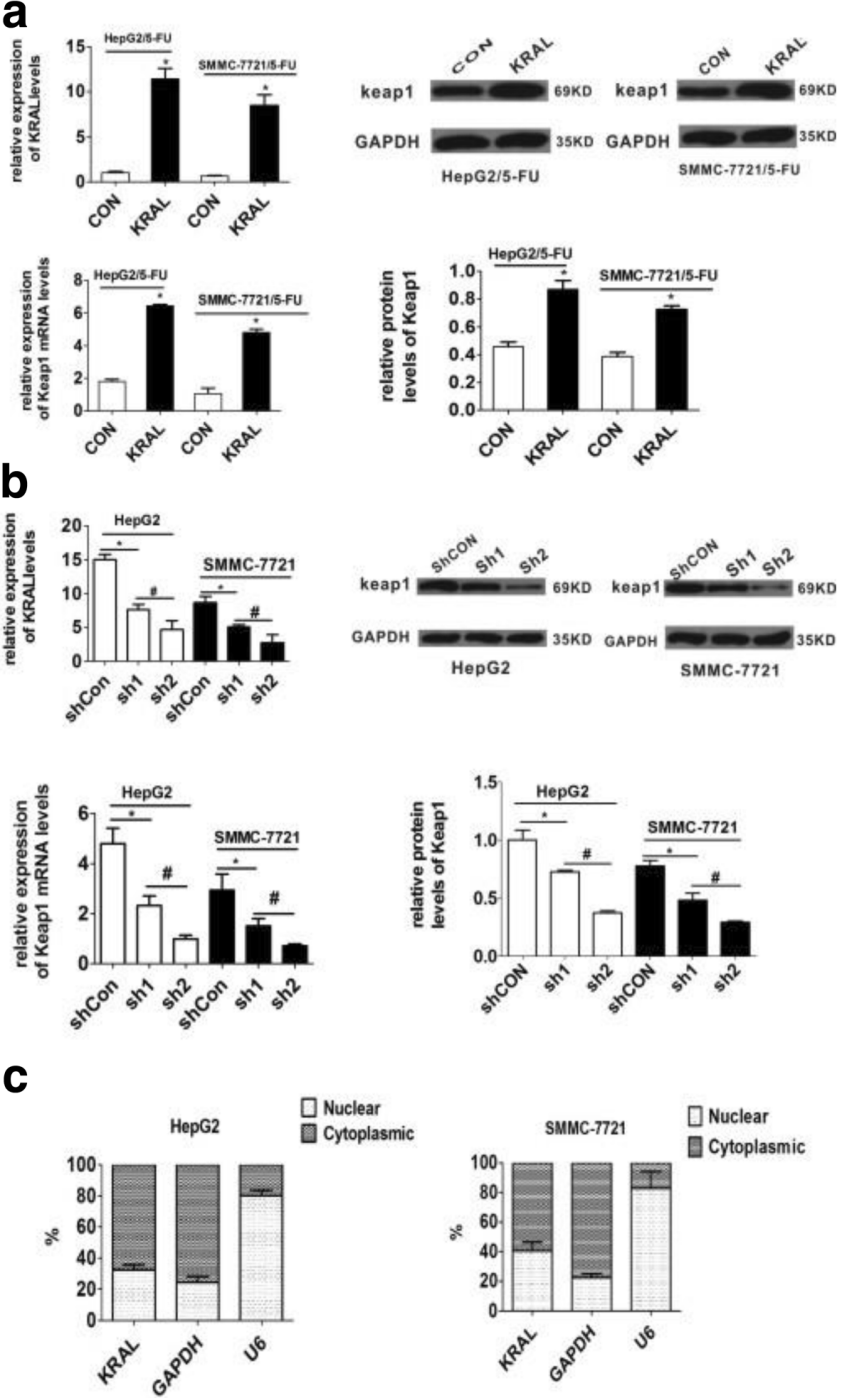
Ectopic expression and knockdown of KRAL. **a** qRT-PCR data showing the relative expression of KRAL in HepG2/5-FU and SMMC-7721/5-FU cells 48 h after transfection with lentivirus expressing empty vector or KRAL, *p < 0.05. The mRNA and protein levels of Keap1 in control and KRAL-overexpressing cells were determined by RT-PCR and western blotting, respectively. GAPDH was used as a reference; data are expressed as the mean ± SD; bar graph indicates the normalized values from at least 3 separate experiments; *p < 0.05 vs control group. **b** qRT-PCR data showing the relative expression of KRAL in HepG2 and SMMC-7721 cells 48 h after transfection with lentivirus expressing shRNA against scramble or KRAL, *p < 0.05, ^*#*^*p* < 0.05. The mRNA and protein levels of Keap1 in control and KRAL knockdown cells were determined by RT-PCR (*p < 0.05, ^*#*^*p* < 0.05) and western blotting (*p < 0.05, ^*#*^*p* < 0.05), respectively. GAPDH was used as a reference; data are expressed as the mean ± SD; bar graph indicates the normalized values from at least 3 separate experiments. **c** Cellular fractions were isolated from HepG2 and SMMC-7721 cells. KRAL was mainly distributed in the cytoplasm. GAPDH mRNA and U6 RNA were used as controls for the cytoplasmic and nuclear RNA fractions, respectively. Data are expressed as the mean ± SD; columns: mean of three independent experiments

As expression of KRAL and Keap1 showed similar changes after 5-FU treatment, it was hypothesized that Keap1 expression may be regulated by KRAL. To confirm this idea, KRAL was stably overexpressed in HepG2/5-FU and SMMC-7721/5-FU cells (Fig. 4a). The results indicated that overexpression of KRAL could markedly enhance the mRNA and protein levels of Keap1 (Fig. 4a). Conversely, silencing KRAL in HepG2 and SMMC-7721 cells significantly suppressed Keap1 expression (Fig. 4b).

To discover the mechanism responsible for KRAL regulation, cellular fractionation was performed to detect the subcellular location of KRAL in HepG2 and SMMC-7721 cells. The data showed that KRAL is mainly located in the cytoplasm of HCC cells, implying a potential role of KRAL in post-transcriptional modulation (Fig. 4c).

### KRAL directly interacts with miR-141

**Fig. 5:**
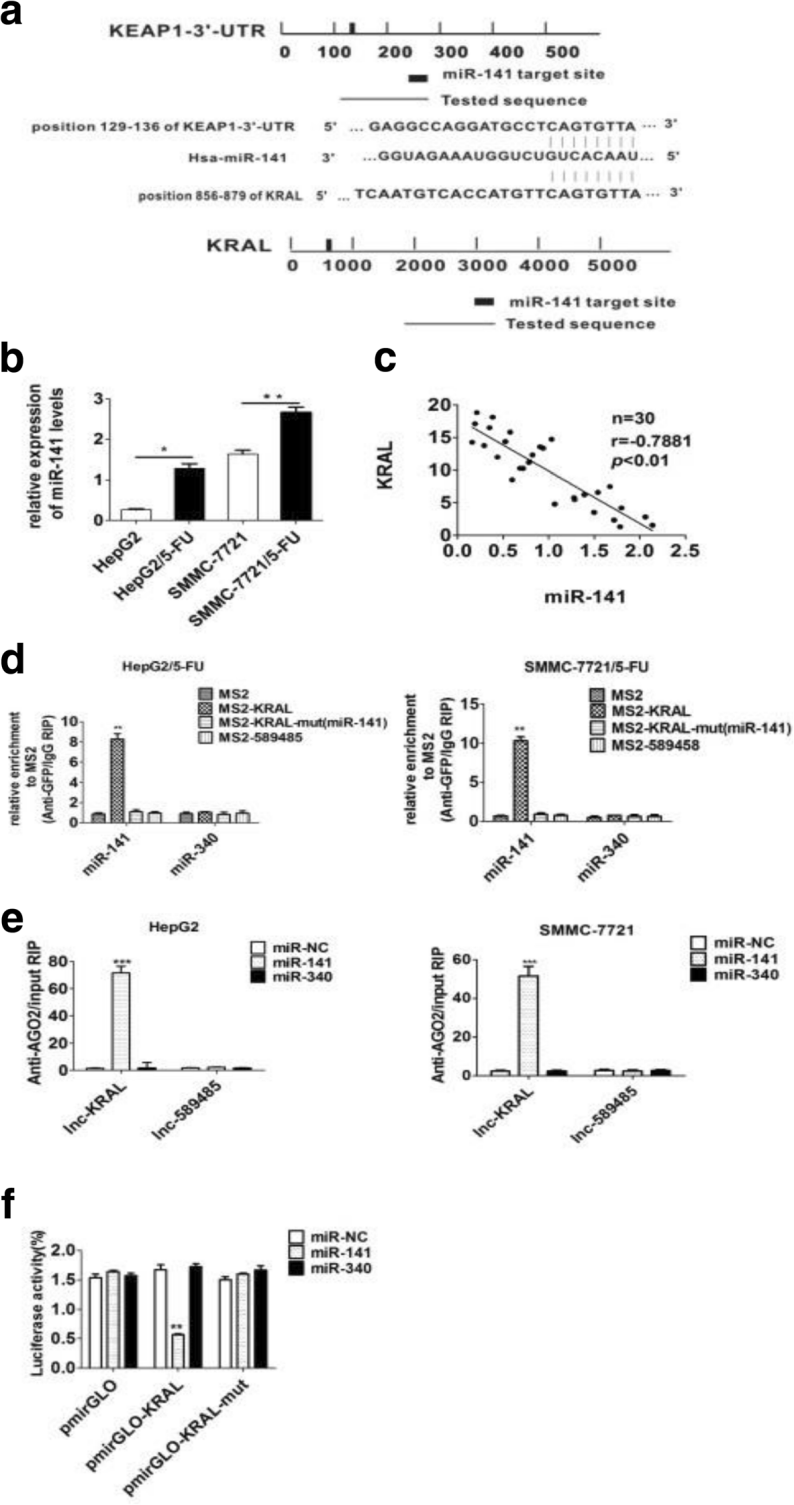
KRAL directly interacts with miR-141. **a** Schematic diagram of the miR-141 binding site in the KRAL and Keap1 3’UTRs based on starBase v.2.0. **b** qRT-PCR data showing the relative expression of miR-141 in HepG2/5-FU and SMMC-7721/5-FU cells compared to that in their respective parent cells. U6 was used as a reference Data are expressed as the mean ± SD; columns: normalized mean of three independent experiments; *p < 0.05, **p < 0.01. **c** Correlation between miR-141 and KRAL expression in 30 HCC tissue samples was assessed by pearson correlation analysis (*r* = − 0.7881, *p* < 0.01). **d** MS2-RNA immunoprecipitation (RIP) followed by qRT-PCR was performed to analyse endogenous miRNAs associated with KRAL; **p < 0.01 vs *the other group.* **e** Anti-AGO2 RIP was performed in HepG2 and SMMC-7721 cells transiently transfected with miR-141, miR340 or miR-NC. The amount of KRAL enriched in the miR-transfected cells was detected by qRT-PCR, GAPDH was used as a reference, Data are expressed as the mean ± SD; columns: normalized mean of three independent experiments; ***p < 0.001 vs *the other group.* **f** Luciferase activity in HEK293T cells co-transfected with miR-141 and luciferase reporters containing nothing (pmirGLO), KRAL, or mutant KRAL (KRAL-mut). Data are presented as the relative ratio of firefly luciferase activity to Renilla luciferase activity. Data are expressed as the mean ± SD, columns: mean of three independent experiments,**p < 0.01 vs *the other group*

Recently, emerging evidence showed that lncRNAs could act as a competitive endogenous RNA (ceRNA) through its modified complementary sequence to microRNAs (miRNAs). To explore whether KRAL could serve as a ceRNA, the software starBase v.2.0 was adopted to predict the potential binding ability of miRNAs to both Keap1–3’UTR and KRAL (Fig. 5a), and a set of candidate miRNAs were obtained. Among these miRNAs, miR-141 was selected for its activity in enhancing 5-FU resistance in HCC cells [8]. Moreover, the qPCR results indicated that miR-141 was significantly increased in 5-FU-resistant HCC cells (Fig. 5b). KRAL levels were negatively associated with miR-141 levels in 30 HCC tissue samples (Fig. 5c).

To confirm the direct binding between miR-141 and KRAL at endogenous levels, RNA immunoprecipitation (RIP) was performed to pull down endogenous miRNAs associated with KRAL. qPCR analysis demonstrated that KRAL RIP in HepG2/5-FU and SMMC-7721/5-FU cells was significantly enriched for miR-141 (but not for miR340) compared to the levels in cells transfected with empty vector (MS2), KRAL mutations at the miR-141 targeting sites (KRAL–mut), or another lncRNA-ENST00000589485 (henceforth named lncRNA-589,485), which is also inhibited by 5-FU but does not have a predicted miR-141 binding site (Fig. 5d).

To determine whether KRAL was modulated by miR-141 in an AGO2-dependent manner, the RIP assay was performed using antibodies against AGO2 in HepG2 and SMMC-7721 cells transiently overexpressing miR-141, miR-NC or miR-340. qRT-PCR was performed to determine RNA levels after immunoprecipitation. The amount of endogenous KRAL pulled down by AGO2 was preferentially enriched in miR-141 overexpressed cells (Fig. 5e), supporting the notion that miR-141 is a KRAL-targeting miRNA.

For further confirmation, luciferase vectors containing wild-type and mutant KRAL (mutations at the miR-141 binding sites) were constructed. The results of the dual-luciferase assays show that co-transfection of miR-141 mimics with the wild-type KRAL vector (pmirGLO-WT-KRAL) but neither empty vector nor the mutant pmirGLO-mut-KRAL vector significantly reduced the luciferase activities (Fig. 5f).

### KRAL functions as a ceRNA of Keap1

**Fig. 6:**
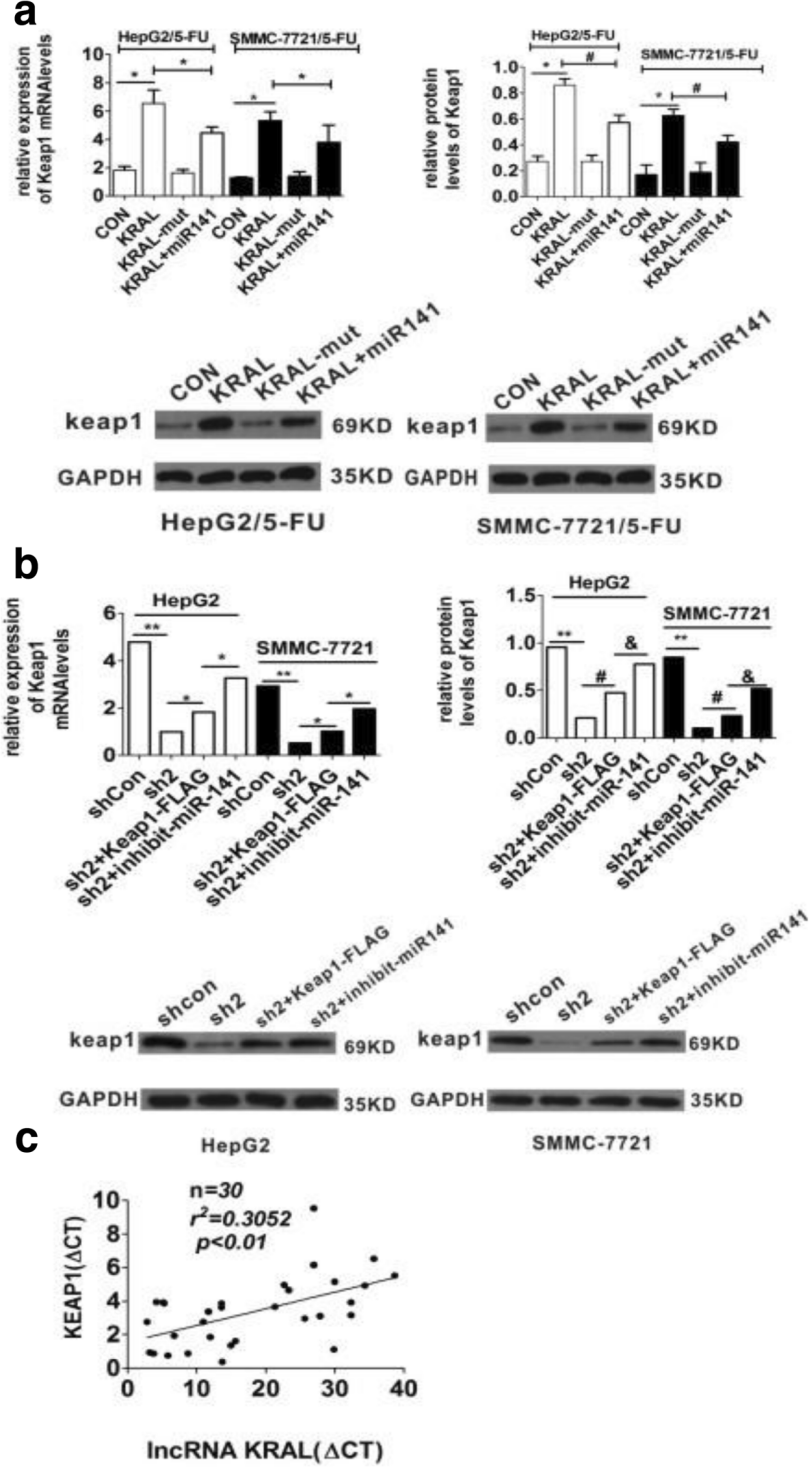
KRAL functions as a ceRNA against Keap1. **a** HepG2/5-FU and SMMC-7721/5-FU cells were transfected with KRAL or KRAL-mut plasmids in the presence or absence of miR141 mimics. qRT-PCR and western blotting were performed to analyse the mRNA (*p < 0.05) and protein levels (*p < 0.05 vs the con group, ^*#*^*p* < 0.05 vs the KRAL group) of Keap1, respectively, GAPDH was used as a reference; data are expressed as the mean ± SD, bar graph indicates the normalized values from at least 3 separate experiments. **b** HepG2 and SMMC-7721 cells were transfected with sh2-KRAL or sh2 -KRAL+Keap1-FLAG plasmids in the presence or absence of miR141 inhibitors. qRT-PCR and western blotting were performed to analyse the mRNA (*p < 0.05, **p < 0.01) and protein levels (*p < 0.05 vs the shcon group, ^*#*^*p <* 0.05 vs the sh2 group, ^&^*p* < 0.05 vs the sh2 + Keap1-FLAG) of Keap1, respectively, GAPDH was used as a reference; data are expressed as the mean ± SD; bar graph indicates the normalized values from at least 3 separate experiments. **c** The correlation between the levels of KRAL and Keap1 mRNA in 30 HCC tissue samples was assessed with the pearson correlation analysis

Previously, it was validated that miR-141 could regulate its binding site within the Keap1 mRNA. Furthermore, overexpression of miR-141 significantly decreased the mRNA and protein levels of Keap1 [8]. Because KRAL served as a sponge for miR-141 in HCC cell lines, it has been speculated that this gene could effectively modulate Keap1 by competitively binding miR-141. To validate this hypothesis, HepG2/5-FU and SMMC-7721/5-FU cells were co-transfected with either wild-type KRAL or mutant KRAL or wild-type KRAL+miR-141 mimics. The mRNA and protein expression of Keap1 was increased with wild-type KRAL overexpression, but not KRAL-mutant overexpression (Fig. 6a). The result also demonstrated that miR-141 mimics could partly abolish the KRAL-induced increase in Keap1 expression (Fig. 6a). In contrast, HepG2 and SMMC-7721 cells were either co-transfected with sh2-KRAL or sh2-KRAL+Keap1-FLAG plasmids or sh2-KRAL + miR141-inhibitors. The results show that Keap1 mRNA and protein expression Keap1was decreased with KRAL knockdown, and Keap1-FLAG plasmids or miR-141 inhibitors could partly restore Keap1 expression (Fig. 6b). Moreover, KRAL expression was positively correlated with Keap1 expression in 30 HCC tissue samples (Fig. 6c).

**Fig. 7:**
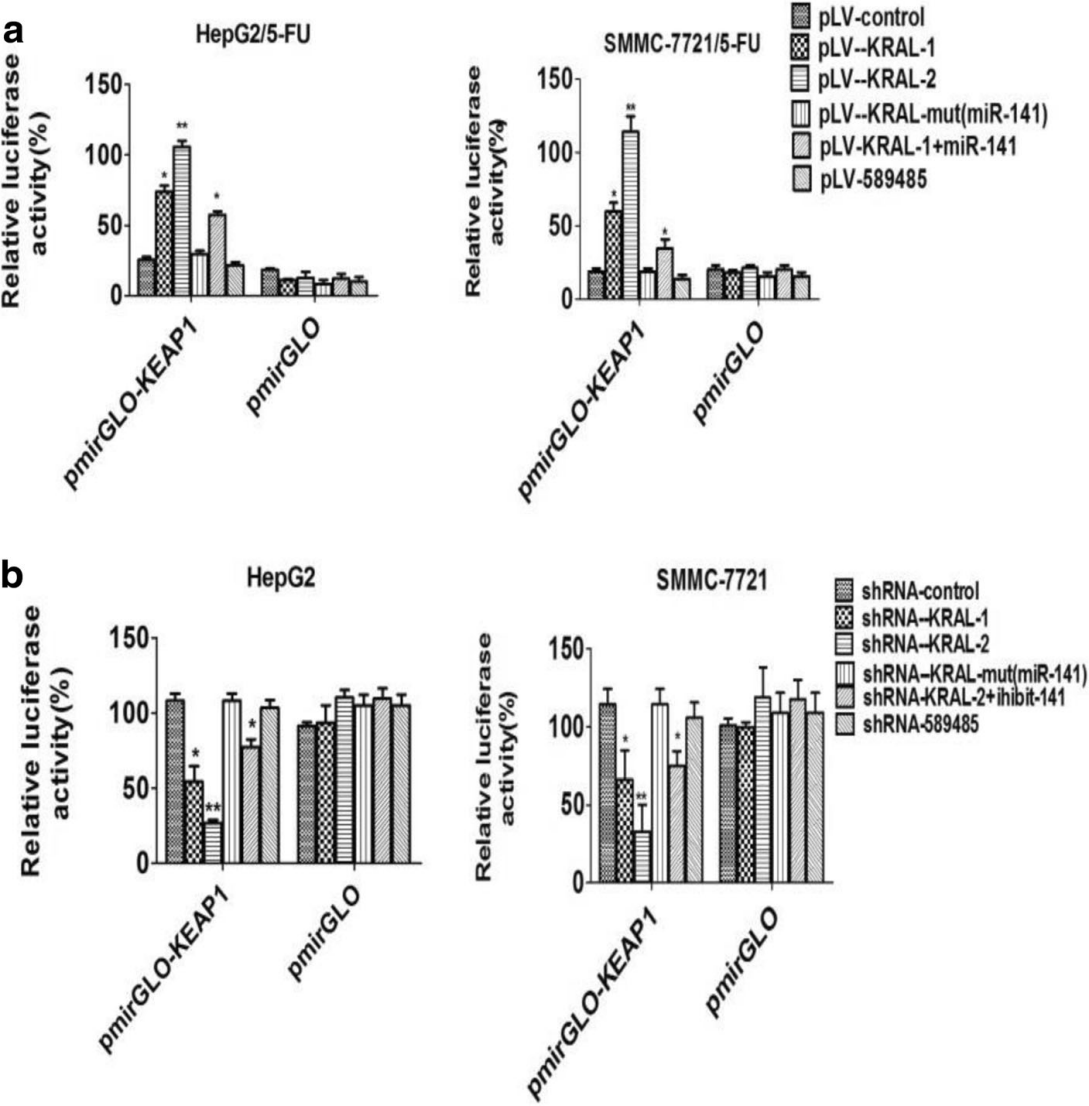
Dual-luciferase reporter assay. **a** HepG2/5-FU and SMMC7721/5-FU cells were co-transfected with plasmids overexpressing wild-type or mutant KRAL and luciferase reporter vector containing either Keap1 3’UTR or empty vector (pmirGLO). Relative luciferase activity was presented as the relative ratio of firefly luciferase activity to Renilla luciferase activity. Data are expressed as the mean ± SD; columns: normalized mean of three independent experiments,*p < 0.05 vs the pLV-control group or pLV-KRAL-mut group, **p < 0.01 vs the KRAL1 group or pLV-KRAL-mut group. **b** HepG2 or SMMC7721 cells were co-transfected with shRNA-KRAL or mutant KRAL plasmids and luciferase reporter vector containing either the Keap1 3’UTR or empty vector (pmirGLO). Relative luciferase activity was presented as the relative ratio of firefly luciferase activity to Renilla luciferase activity. Data are expressed as the mean ± SD; columns: mean of three independent experiments; *p < 0.05 vs the pLV-control group or pLV-KRAL-mut group, **p < 0.01 vs the shRNA-KRAL-1 group or pLV-KRAL-mut group

Subsequently, to explore whether KRAL regulated Keap1 expression through the regulation of Keap1–3’UTR, the pmirGLO-Keap1–3’UTR plasmid was co-transfected with the pLV-KRAL1/2 plasmids, shR-KRAL-1/2 plasmids, and either miR-141 mimics or miR-141 inhibitor in resistant HCC cells and their parental strains, respectively. Overexpression of wild-type KRAL, but not mutant KRAL or lncRNA-589,485, increased the luciferase activity of pmirGLO-Keap1 in a dose-dependent manner. Ectopic expression of miR-141 abolished this upregulation. The result indicated that KRAL blocked miR-141 and released Keap1 from miR-141. Reciprocally, the data also showed that knocking down wild-type KRAL, but not mutant KRAL or lncRNA-589,485, significantly decreased luciferase activity; this decrease was prevented by miR-141 inhibitors, further suggesting that KRAL and Keap1 engage in crosstalk by competing for miR-141 binding (Fig. 7a-b).

### KRAL reverses 5-fluorouracil resistance in HCC cell lines by regulating Keap1 expression

**Fig. 8:**
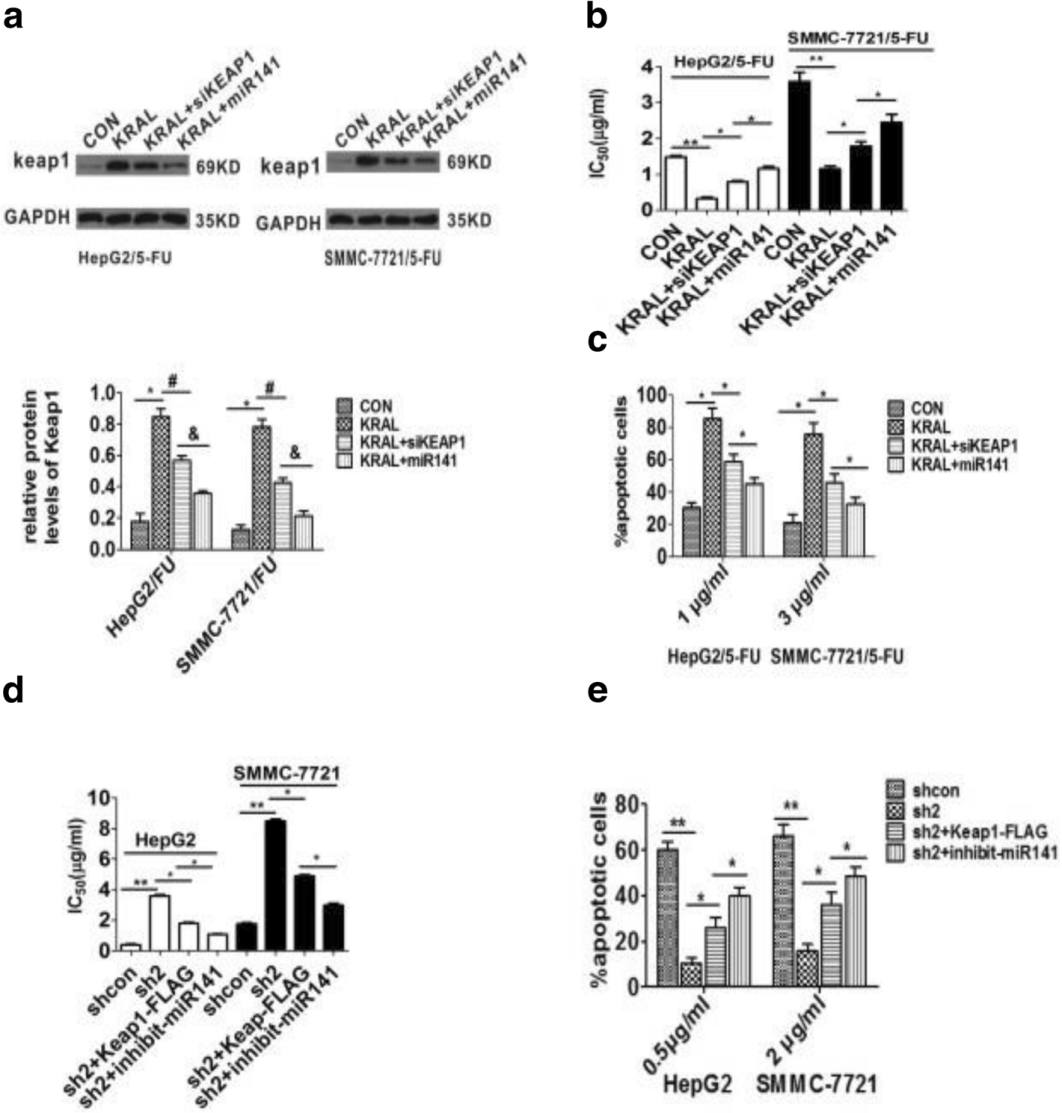
KRAL reverses 5-FU resistance in HCC cell lines by regulating Keap1 expression. **a** Western blotting was performed to detect Keap1 expression in KRAL-overexpressing HepG2/5-FU and SMMC-7721/5-FU cells transfected with Keap1 siRNA or miR-141 mimics. GAPDH was used as a reference; data are expressed as the mean ± SD; bar graph indicates the normalized values from at least 3 separate experiments; *p < 0.05, ^*#*^*p <* 0.05, ^&^*p* < 0.05. **b** The IC_50_ of 5-FU in KRAL-overexpressing HepG2/5-FU and SMMC-7721/5-FU cells transfected with Keap1 siRNA or miR-141 mimics. Data are expressed as the mean ± SD; columns: mean of three independent experiments; *p < 0.05, **p < 0.01. **c** Transfected HepG2/5-FU and SMMC-7721/5-FU cells were exposed to the indicated doses of 5-FU for 48 h. The cells were stained with annexin-V/PI and subjected to flow cytometry. Bar graph indicates the relative percentages of apoptotic cells from three independent experiments, *p < 0.05. **d** The IC_50_ of 5-FU in KRAL-silenced HepG2 and SMMC-7721 cells transfected with Keap1-FLAG plasmids or miR-141 inhibitor. Data are expressed as the mean ± SD; columns: mean of three independent experiments; *p < 0.05, **p < 0.01. **e** KRAL-silenced HepG2 and SMMC-7721 cells were exposed to the indicated doses of 5-FU for 48 h. The cells were stained with annexin-V/PI and subjected to flow cytometry. Bar graph indicates the relative percentages of apoptotic cells from three independent experiments. *p < 0.05, **p < 0.01

Apoptosis is an important mechanism involved in 5-FU chemotherapy. Therefore, downregulation of KRAL may play a key role in the development of drug resistance by impairing 5-FU-induced apoptosis. To validate this hypothesis, HepG2/5-FU and SMMC-7721/5-FU cells were transfected with pLV-KRAL or pLV-KRAL+miR-141 mimics or negative controls and incubated with different concentrations of 5-FU. The results indicated that compared with control cells, cells with KRAL overexpression exhibited a reversal in the resistance against 5-FU, with a significant decrease in the IC_50_ and a dramatic increase in cellular apoptosis, while silencing Keap1 or ectopically expressing miR-141 partially rescued this effect (Fig. 8a-c). In addition, compared with their respective control cells, KRAL-silenced HepG2 and SMMC-7721 cells showed much higher 5-FU resistance, with a prominent increase in the IC_50_ value and a marked decrease in cellular apoptosis, while ectopically expressing Keap1 or silencing miR-141 partially abolished this promotion induced by KRAL knockdown (Fig. 8d-e). Collectively, these data suggest that KRAL reverses 5-FU resistance in HCC cell lines by regulating Keap1 expression.

### KRAL inhibits the Nrf2 pathway by regulating Keap1 expression in a dose-dependent manner

**Fig. 9:**
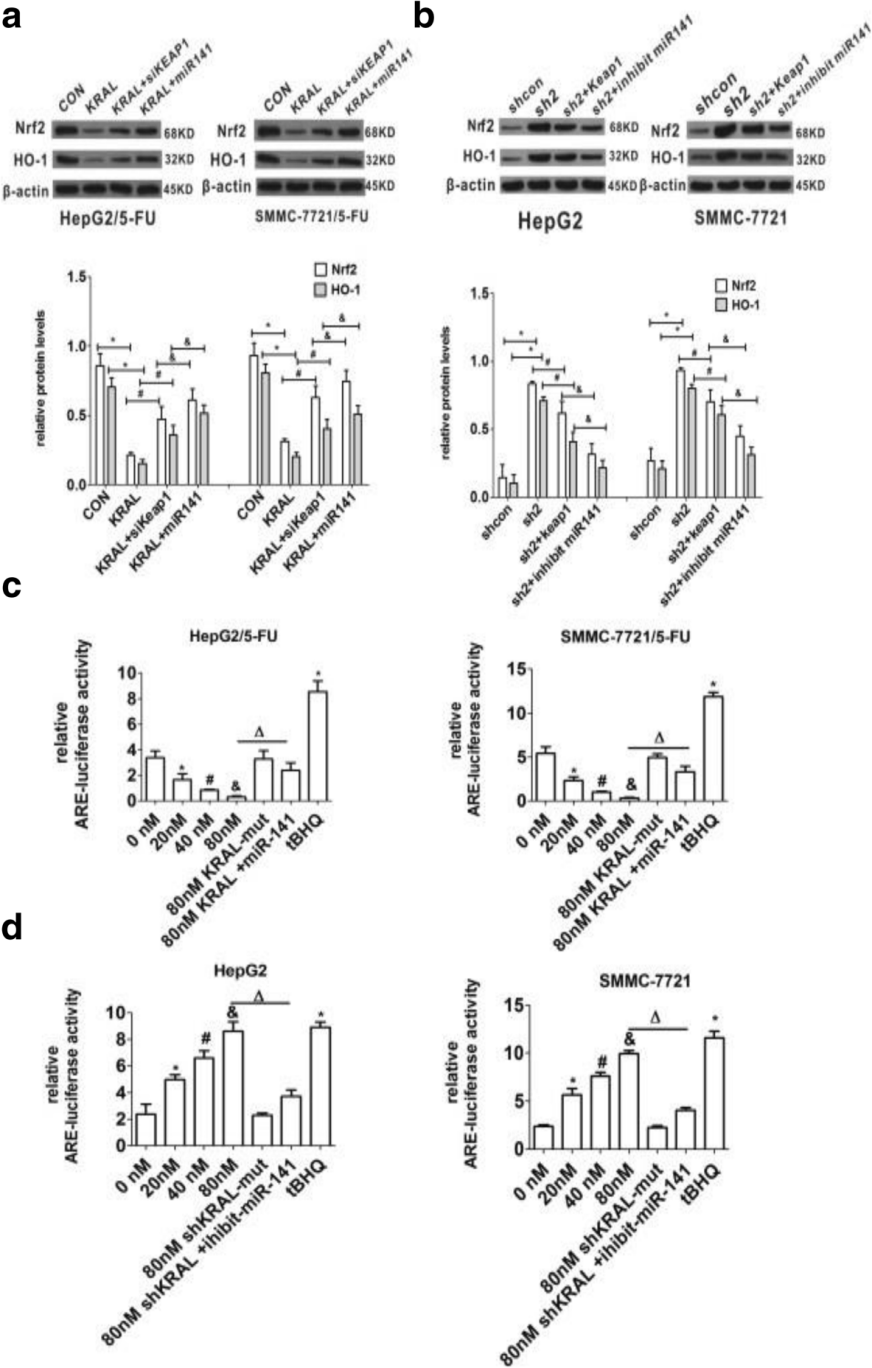
KRAL inhibits the Nrf2 pathway by regulating Keap1 expression in a dose-dependent manner. **a** Western blotting was performed to detect the protein levels of Nrf2 and HO-1 in KRAL-overexpressing HepG2/5-FU and SMMC-7721/5-FU cells transfected with Keap1 siRNA or miR-141 mimics, GAPDH was used as a reference; data are expressed as the mean ± SD; bar graph indicates the normalized values from at least 3 separate experiments; *p < 0.05 vs the CON group, ^*#*^*p <* 0.05 vs the KRAL group, ^&^*p* < 0.05 vs the KRAL+siKEAP1 group**. b** Western blotting was performed to detect the protein levels of Nrf2 and HO-1 in KRAL-silenced HepG2 and SMMC-7721 cells transfected with Keap1-FLAG plasmids or miR-141 inhibitor. GAPDH was used as a reference; data are expressed as the mean ± SD; bar graph indicates the normalized values from at least 3 separate experiments; *p < 0.05 vs the shcon group, ^*#*^*p <* 0.05 vs the sh2 group, ^&^*p* < 0.05 vs the sh2 + Keap1 group. **c** HepG2/5-FU and SMMC-7721/5-FU cells were transiently transfected with HO-1-ARE-luciferase plasmid or control vector and then transfected with different concentrations of KRAL or KRAL-mut plasmids in the presence or absence of miR-141 mimics; tBHQ was used as a positive control. The induced fold change in luciferase activity for cell lysates was analysed by normalizing the transfection efficiency and dividing the values of each experiment to those of the control. Data are expressed as the mean ± SD; columns: normalized mean of three independent experiments; *p < 0.05 vs the 0 nm group, ^*#*^*p <* 0.05 vs the 20 nm group, ^&^*p* < 0.05 vs the 40 nm group, ^Δ^*p* < 0.05 vs the 80 nm group. **d** HepG2 and SMMC-7721 cells were transiently transfected with HO-1-ARE-luciferase plasmid or control vector and then transfected with different concentrations of shRNA-KRAL or KRAL-mut plasmids in the presence or absence of miR-141 inhibitors; tBHQ was used as a positive control. The induced fold change in luciferase activity for cell lysates was analysed by normalizing the transfection efficiency and dividing the values of each experiment relative to those of the control. Data are expressed as the mean ± SD; columns: mean of three independent experiments; *p < 0.05 vs the 0 nm group, ^*#*^*p <* 0.05 vs the 20 nm group, ^&^*p* < 0.05 vs the 40 nm group, ^Δ^*p* < 0.05 vs the 80 nm group

Western blotting was performed to detect whether the Nrf2 pathway could be modulated by KRAL. Total Nrf2 and HO-1 were decreased in HepG2/5-FU and SMMC-7721/5-FU cells transfected with pLV-KRAL compared to the levels in negative control cells, while silencing Keap1 or ectopically expressing miR-141 partially rescued this effect (Fig. 9a). Conversely, total Nrf2 and HO-1 were increased in HepG2 and SMMC-7721 cells transfected with sh-KRAL-1/2 plasmids compared to the levels in the corresponding negative control cells, while ectopically expressing Keap1 or silencing miR-141 partially diminished KRAL knockdown-induced increases in total Nfr2 and HO-1 expression (Fig. 9b).

To further validate whether KRAL inhibits the Nrf2 pathway by regulating Keap1 expression in a dose-dependent manner, parental and resistant HCC cells were co-transfected with HO-1-ARE luciferase plasmid or control vector (10 ng) and shR-KRAL-1/2 plasmids or pLV-KRAL plasmids, combined either miR-141 inhibitor or miR-141 mimics, respectively. In KRAL-overexpressed HepG2/5-FU and SMMC-7721/5-FU cells, ARE-driven luciferase activity decreased in a dose-dependent manner, and this effect can be partly blocked by miR-141 mimics (Fig. 9c). ARE-driven luciferase activity increased in a dose-dependent manner in KRAL-silenced HepG2 and SMMC-7721 cells, and this effect can be partly blocked by miR-141 inhibitor (Fig. 9d).

## Discussion

5-FU is a classic chemotherapeutic drug that is widely used to treat numerous cancers, including HCC. However, the majority of HCC patients exhibit primary or acquired drug resistance during 5-FU chemotherapy, which greatly limits the clinical applications of 5-FU. Despite advancements in biological technologies in the last several decades, the precise molecular mechanisms involved in 5-FU resistance remain largely unexplored. Abnormal regulation of the Keap1-Nrf2-ARE signalling axis is considered as a major contributor to drug resistance.

In the present study, it was discovered that a gradual decrease in Keap1 expression and increase in total Nrf2 were accompanied by an increase in 5-FU resistance in HepG2/5-FU and SMMC-7721/5-FU cells (Fig. 1a-d) [8], which further supports this viewpoint. Therefore, uncovering the molecular mechanism of Keap1 downregulation would be helpful for overcoming 5-FU resistance in HCC cells.

Numerous reports have demonstrated that lncRNAs play functional roles in regulating multidrug resistance of cancer cells [10, 11]. The lncRNA H19, for example, confers cisplatin resistance in high-grade advanced ovarian tumours [11], Overexpression of the lncRNA LINC00161 enhanced cisplatin-induced apoptosis in osteosarcoma cells by modulating the miR-645-IFIT2 axis [12], the lncRNA SLC25A25-AS1 has been reported to mediate drug resistance and EMT in colorectal cancer cells [9], and linc-ROR exhibited impaired sensitivity to 5-FU in breast cancer cells [13]. Here, the current finding identified a set of differentially expressed lncRNAs in 5-FU-resistant HCC cells, the majority of which, including RP11-65 N13.7, AC144835.1, XLOC_007556, and ENSG00000224386, exhibited the properties of oncogenes [11]. 5-FU resistance is a malignant phenotype; therefore, the result of the current lncRNA assay is in the agreement with previous reports, which validate the profiling observations. However, the exact mechanism by which lncRNAs influence 5-FU resistance in HCC cells is unclear.

In the present study, the results demonstrated that decreased KRAL and Keap1 expression were positively associated with augmented 5-FU resistance (Fig. 1a-c). Further investigation demonstrated that KRAL regulates Keap1 expression by completely sponging miR-141, which inhibited the miR-141-mediated degradation of Keap1 mRNA. To the best of our knowledge, the current research is the first to demonstrate that KRAL works as a ceRNA against Keap1 to sponge and suppress miR-141. Ectopic expression of KRAL could reverse 5-FU resistance in HCC cells, while silencing Keap1 or overexpressing miR-141 partially rescued this effect (Figs. 6, 7, 8). The results indicated that other mechanisms may also participate in KRAL-mediated 5-FU resistance.

The Keap1-Nrf2 signalling axis plays a critical role in cytoprotective responses against electrophiles and oxidative stress [14]. The interaction of Nrf2 with Keap1 results in the degradation of Nrf2, which mediated by the ubiquitin-proteasome pathway. In the presence of electrophiles and oxidative stress, Nrf2 dissociates from Keap1 and translocates into the nucleus, thus activating the downstream phase II detoxifying enzymes and antioxidant proteins. Emerging evidence has demonstrated that activation of Nrf2 activity confers resistance to radio- and chemotherapies onto cancer cells [15]. Mechanistically, the current result also demonstrated that KRAL overexpression could increase the Keap1 levels and impair the expression of genes downstream of the Nrf2 pathway, thus reversing the resistance of HCC cells to 5-FU. Furthermore, these effects were partly abolished by siRNA-Keap1 or overexpression of miR-141 (Fig. 9a). In contrast, silencing KRAL could decrease the Keap1 levels and enhance the expression of genes downstream of the Nrf2 pathway, thus promoting 5-FU resistance in HCC cells, while ectopically expressing Keap1 or silencing miR-141 partially abolished KRAL knockdown-mediated changes (Fig. 9b).

Furthermore, the HO-1-ARE luciferase assay validated that KRAL could inhibit the Nrf2 pathway in HepG2/5-FU and SMMC-7721/5-FU cells by regulating Keap1 expression Keap1 in a dose-dependent manner, and this effect can be partly blocked by miR-141 mimics (Fig. 9c). Conversely, ARE-driven luciferase activity in KRAL-silenced HepG2 and SMMC-7721 cells increased in a dose-dependent manner, and this effect can be partly blocked by miR-141 inhibitor (Fig. 9d).

## Conclusions

In summary, for the first time, KRAL was discovered as a novel critical regulator of Keap1 for mediating 5-FU resistance in HCC cells. KRAL competes with the 3’UTR of Keap1 mRNA to bind miR-141; this competition promotes Keap1 expression and inhibits the Nrf2-ARE pathway, thus leading to a reversal of 5-FU resistance in HCC cells. Because of this crucial role of KRAL in 5-FU drug resistance, this KRAL/miR-141/Keap1 axis holds great promise as a potential therapeutic target for overcoming 5-FU resistance in HCC cells.

## Abbreviations

5-FU: 5-fluorouracil
ANOVA: one way analysis of variance
CCK-8: Cell counting kit
ceRNA: Competitive endogenous RNA
HCC: Hepatocellular carcinoma
Keap1: Kelch-like ECH-associated protein
KRAL: Keap1 regulation associated lncRNA
lncRNA: Long non-coding RNA
Nrf2: Nuclear factor erythroid 2-related factor 2
qRT-PCR: Real-time quantitative reverse transcription PCR
RIP: RNA Immunoprecipitation
SDS-PAGE: Sodium dodecyl sulphate-polyacrylamide gel electrophoresis
